# AGING IN CROSS-NATIONAL CONTEXTS

**DOI:** 10.1093/geroni/igac059.1497

**Published:** 2022-12-20

**Authors:** Jennifer Ailshire, Eunyoung Choi

## Abstract

Cross-national comparative research is a useful tool for identifying common aspects of, and risk factors for, healthy and unhealthy aging across populations and sociocultural contexts. The papers in this symposium use harmonized data from the Gateway to Global Aging to examine a range of topics in aging and provide new insights into long-standing and emerging questions in aging research. Using data on 31 countries, Ehrlich estimates the population attributable fraction of dementia due to vision impairment, a treatable and thus potentially viable target for interventions to slow progression to dementia. Seligman et al., use a frailty index validated in multiple countries to provide new evidence for which aspects of socioeconomic status (SES) – education, income, rural residence – are most strongly linked to frailty in Brazil, China, and India, three highly populated and rapidly aging countries. Using recent harmonized data on stress, Chen et al. examine the association between cumulative social stressors and cognitive function trajectories in the US and UK, finding interesting patterns in the relationship with status and change over time. Finally, several harmonized aging studies collected data throughout the COVID-19 pandemic, and Mair et al., take advantage of this to gain insights into how family structure impacted experiences of loneliness among older adults during the pandemic in the U.S. and across Europe. The papers in this symposium demonstrate the tremendous potential for using cross-national comparisons to deepen our understanding of health and well-being among older adults.

